# Ineffective Esophageal Motility with and Without Abnormal Acid Exposure: Clinical, Endoscopic, and Manometric Features

**DOI:** 10.5152/tjg.2026.25627

**Published:** 2026-07-27

**Authors:** Harun Erdal, Yusuf Coşkun, Deniz Armağan Deniz, Burak Birsoy, Yusuf Üstün

**Affiliations:** 1 Department of Gastroenterology, Ankara Etlik City Hospital, University of Health Sciences, Ankara, Türkiye

**Keywords:** Esophageal acid exposure, gastroesophageal reflux disease, high-resolution manometry, impedance, ineffective esophageal motility, pH metry

## Abstract

**Background/Aims:**

Ineffective esophageal motility (IEM) is the most common esophageal motility disorder. Reflux is common in patients with IEM. The relationship between IEM and gastroesophageal reflux disease is not fully understood. The current study evaluated the associations between clinical, endoscopic, and manometric characteristics and acid exposure in patients with IEM.

**Materials and Methods:**

The authors prospectively enrolled patients diagnosed with IEM who underwent high-resolution manometry according to the Chicago classification, version 4.0. Patients were classified into pathological and physiological reflux groups based on multichannel intraluminal impedance-pH ( MII-pH) monitoring results. Clinical, endoscopic, manometric, and MII-pH parameters were compared between the 2 groups.

**Results:**

Of the 69 patients, 49 (71%) had pathological reflux and 20 (29%) had physiological reflux. The most frequent symptoms were heartburn, regurgitation, and dysphagia. No significant differences were observed between the 2 groups in demographic, clinical, or endoscopic characteristics. Among the manometric metrics, mean lower esophageal sphincter pressure and esophagogastric junction (EGJ) morphology differed significantly between the 2 groups (*P* = .017 and *P* = .046, respectively). All MII‑pH metrics, except weak acid reflux episodes and nonacid reflux episodes, differed significantly between the groups (*P* <.05).

**Conclusion:**

Acid exposure did not affect symptoms in patients with IEM. In addition to impaired esophageal body contractility, impaired esophageal clearance and EGJ barrier dysfunction may contribute to acid exposure.

**Main Points**

- Pathological reflux is common in patients with ineffective esophageal motility (IEM). However, patients with IEM present with diverse symptoms.
- In addition to weak and failed peristalsis, impaired esophageal clearance and esophagogastric junction (EGJ) barrier dysfunction contribute to acid exposure and symptoms generation in patients with IEM.
- Mean lower esophageal sphincter pressure was significantly lower in the pathological reflux group than in the physiologic reflux group. The type III EGJ subtype was significantly more prevalent in the pathological reflux group.

## Introduction

Ineffective esophageal motility (IEM) is the most common esophageal motility disorder.^1^ The Chicago Classification v.4.0 (CC v4.0) established the latest definition of IEM. According to CC v4.0, a definitive diagnosis of IEM requires more than 70% ineffective swallows or at least 50% failed peristaltic sequences. An ineffective swallow is defined as a weak contraction (distal contractile integral (DCI) ≥ 100 mmHg.s.cm and < 450 mmHg.s.cm), failed peristalsis (DCI < 100 mmHg.s.cm), or a fragmented swallow.^2^ Gastroesophageal reflux disease (GERD) is associated with impaired esophageal clearance, which is believed to contribute to its pathophysiology.^3^ Pathological acid reflux is more common in patients with IEM than those with normal esophageal motility.^4^^,^^5^ Symptoms in patients with IEM vary widely. Some patients are asymptomatic, whereas others present with dysphagia, regurgitation, or heartburn. However, the pathophysiology of IEM remains unclear.^1^ Previous studies have examined the relationship between GERD and IEM. In patients with GERD, esophageal muscle contraction may be impaired by acid exposure, leading to increased acid exposure and more severe symptoms.^6^Several studies have demonstrated that the duration of pathological acid exposure correlates with the presence of IEM.^7^ Additionally, IEM can occur in patients without evidence of GERD findings, and some studies have found no significant association between IEM and GERD.^8^

The primary outcome was the presence of pathological reflux in patients with IEM, defined according to the Lyon Consensus v2.0 criteria. Manometric and multichannel intraluminal impedance-pH (MII-pH) parameters were evaluated as predictors of pathological reflux. Furthermore, the authors examined both pathological and physiological reflux through clinical, endoscopic, manometric, and MII-pH parameters.

## Material and Methods

### Study Design

This single-center, prospective, cross-sectional study enrolled patients evaluated in the gastroenterology motility laboratory of the gastroenterology clinic at the Ankara Etlik City Hospital. The study was conducted in accordance with the Declaration of Helsinki and was approved by the Ankara Etlik City Hospital Ethics Committee (Approval no: 018/2025, Date: November 11, 2024). No Artificial Intelligence tool and/or large language models were used.

### Patient Selection

The study included consecutive adult patients who underwent high-resolution manometry (HRM) at Ankara Etlik City Hospital Gastroenterology Clinic between January 2025 and July 2025. Patients aged 18 years or older who agreed to participate and underwent both HRM and 24-hour MII-pH monitoring were included. Patients aged less than 18 years, those with a history of esophageal or gastric surgery, and those with a non-IEM esophageal motility disorder such as achalasia, esophagogastric junction (EGJ) outflow obstruction, or distal esophageal spasm, as diagnosed by HRM, were excluded. Written informed consent was obtained from all participants.

### High-Resolution Manometry Procedure and Analysis

Water-perfused HRM demonstrated a higher percentage of ineffective swallows than solid-state HRM, potentially resulting in overdiagnosis of IEM disorders.^9^ In this study, HRM was performed using the ManoScan Eso solid-state esophageal manometry catheter and analyzed with ManoView software (Medtronic, Minneapolis, MN, USA). All HRM procedures were performed according to the institution’s standard protocol based on CC v4.0, which consisted of 10 supine 5-mL water swallows administered by syringe after successful catheter placement. Provocative tests and upright swallows were also performed.^10^ However, only the supine swallows were included in the study. According to CC v4.0, the morphology of the EGJ is defined by the relationship between the lower esophageal sphincter (LES) and crural diaphragm (CD). Type I is characterized by complete overlap of the LES and CD, type II by separation of 1 to 2 cm, and type III by separation of more than 2 cm.

Nicodeme et al^11^ introduced the esophagogastric junction contractile integral (EGJ-CI) as a measure of strength of the EGJ. EGJ-CI was calculated using the DCI, which was set to include the LES and CD over 3 respiratory cycles at a specified gastric pressure. The DCI was then divided by the duration of the 3 respiratory cycles to derive the EGJ-CI in mm Hg.^12^

### 24‑Hour Multichannel Intraluminal Impedance‑pH Monitoring

Patients were instructed to discontinue medications that could affect esophageal motor function, such as proton pump inhibitors and histamine H2-receptor antagonists, for at least 7 days before the test. A MII-pH monitoring catheter with Reflux Reader 6.1 analysis software (Medtronic) was used to measure and interpret esophageal intraluminal impedance and pH values. The pH sensor was positioned 5 cm above the LES, and 6 impedance sensors were positioned at 3, 5, 7, 9, 15, and 17 cm above the LES. All participants documented the timing of their symptoms, dietary intake, and changes in body position. Acid exposure time (AET) is defined as the proportion of a 24-hour monitoring period during which the distal esophageal pH is <4. Reflux events were classified as acid reflux (pH <4), weakly acid reflux (pH 4-7), and nonacid reflux (pH >7).^13^ Bolus exposure time (BET) and mean nocturnal baseline impedance (MNBI) were also calculated. The most recent definition of GERD was established according to the Lyon Consensus v2.0.^14^

### Statistical Analysis

Statistical Package for the Social Sciences (SPSS) version 27 (IBM SPSS Corp.; Armonk, NY, USA) was used for data analysis and table generation. Data normality was assessed using either the Kolmogorov–Smirnov test or the Shapiro–Wilk test. Descriptive statistics for all continuous variables are presented as mean ± SD, whereas categorical variables are presented as counts and percentages. For normally distributed data, an independent-samples *t*-test was used. For non-normally distributed data, the Mann–Whitney *U-*test was used. *P* values <.05 were considered significant. Logistic regression analysis was performed to identify predictors of pathological reflux.

The estimated sample size was calculated assuming an effect size of 0.5 and was determined to be at least 45 cases to achieve 95% power and 95% confidence level using G*Power version 3.1.9.4 computer software (Universität Düsseldorf, Germany).

## Results

### Demographic Characteristics

The study group included 69 patients, with an average age of 47.42 ± 16.14 years. Forty patients (58%) were female, and 29 (42%) were male. Patients were categorized into 2 groups according to the Lyon Consensus v2.0 criteria: physiological reflux (AET <4%) and pathological reflux (AET >6%). One patient had an AET of 4%-6% and a total reflux of >80/day. Therefore, this patient was included in the pathological reflux group. Of the 69 patients, 49 (71%) had pathological reflux and 20 (29%) had physiological reflux. The demographic, clinical, and endoscopic features of patients with IEM are summarized in Table 1.

There were no differences between the 2 groups in terms of age, sex, smoking status, and body mass index (BMI). The most common symptoms were heartburn, regurgitation, and dyspepsia. No significant differences were observed between the pathologic and physiologic reflux groups regarding symptoms. Endoscopic findings included normal results in 57 patients and hiatal hernias in 21 patients (82.6% and 30.4%, respectively). There was no significant difference in endoscopic findings between the 2 groups.

### High-Resolution Manometry Metrics in Patients with Ineffective Esophageal Motility

Analysis of HRM metrics showed that type I EGJ morphology (50.7%) was the most common and that the mean lower esophageal sphincter pressure (LESP) was 12.36 mm Hg among patients with IEM. The mean LESP was 11.14 mm Hg in the pathological reflux group and 16.4 mm Hg in the physiological reflux group. Significant differences between the pathological and physiological reflux groups were observed for mean LESP and EGJ morphology (*P* = .017 and *P* = .046, respectively). the pathological reflux group had a significantly lower mean LESP. Type I EGJ morphology was the most common in both groups. However, the type III EGJ was more significant in the pathological reflux group. There were no significant differences between the groups in terms of median integrated relaxation pressure (IRP), mean DCI, EGJ-CI, and peristalsis classification (Table 2).

### Multichannel Intraluminal Impedance-pH Monitoring Metrics in Patients with Ineffective Esophageal Motility

Analysis of MII‑pH metrics showed that the DeMeester score, AET (%), upright AET (%), supine AET (%), BET (%), upright BET (%), supine BET (%), total reflux episodes, prolonged (>5 minutes) reflux episodes, longest reflux time, acid reflux episodes, total reflux episodes, and MNBI at 3 cm (Ω) were significantly higher in the pathological reflux group (*P* ≤ .005). The differences among variables reflect the study’s methodology. No significant differences were found between the groups in weak acid reflux episodes and nonacid reflux episodes (Table 3).

### Multivariate Logistic Regression Analysis of Predictor Factors

Logistic regression analysis identified mean LESP and EGJ morphology as significant predictors of pathological reflux, whereas hiatal hernia, median IRP, mean DCI, and EGJ-CI were not significant (Table 4).

## Discussion

This study demonstrated that pathological reflux is common among patients with IEM and that the most common symptoms are heartburn, regurgitation, and dysphagia. Manometric metrics, such as mean LESP and EGJ morphology, differed significantly between the pathological and physiological reflux groups.

IEM is the most common esophageal motility disorder. It may present with reflux symptoms, especially dysphagia, regurgitation, or heartburn, or it may be asymptomatic. Symptom severity does not correlate with disease severity.^1^ Ho et al^1^,^15^ reported that half of patients with GERD had IEM and that those with Barrett esophagus were more likely to have IEM. Although treatment includes dietary and lifestyle modifications and management of GERD, no effective treatment for IEM is currently available.

The etiology of IEM remains unclear. The cause-and-effect relationship between IEM and GERD has not been clearly established. Although IEM is often associated with typical GERD symptoms, such as heartburn and/or regurgitation, whether GERD and IEM are causally related remains uncertain. Additionally, the direction of this potential causality remains unclear. GERD could cause decreased peristaltic contractility through reflux-induced injury, whereas ineffective esophageal motility (IEM) may predispose patients to GERD by reducing esophageal clearance of refluxate.^16^

The authors found that the pathological reflux group had a higher mean BMI. He et al,^17^ reported that individuals with metabolically healthy obesity were more likely to have GERD and IEM than metabolically healthy individuals without obesity.

GERD and acid exposure increase intercellular spaces in the esophageal epithelium, impair neuronal transmission between muscle cells, and contribute to hypomotility.^1^In a study of patients with PPI-resistant reflux symptoms, esophageal biopsies were obtained to determine whether altered mucosal sensory afferents contribute to the development of IEM. Neither the location of the mucosal sensory afferent nerves nor mRNA levels of potential mechanoreceptors correlated with weak esophageal contractions. IEM is a common motility disorder, and its underlying mechanisms remain unclear.^18^

In this study, the authors examined the relationship between IEM and esophageal reflux burden, as well as clinical, endoscopic, and manometric features. Of the 69 patients with IEM, 71% had pathological reflux, whereas 29% had physiological reflux. The most common symptoms were heartburn, regurgitation, and dysphagia, which are consistent with the existing literature.^5^^,^^10^^,^^19^^,^^20^ However, no significant differences were found between pathological and physiological acid exposures, suggesting that the symptoms are not solely linked to acid exposure burden. It is important to note that some patients with IEM and GERD symptoms may not show improvement with acid inhibition therapy. As outlined in the Lyon Consensus, the diagnosis of GERD was established using MII-pH monitoring.

In a study evaluating the relationship between IEM and symptom severity, investigators hypothesized that a higher proportion of weak or failed swallows, as assessed by peristaltic contractility, would be associated with more severe dysphagia symptoms. However, patients with IEM did not differ from those with normal HRM findings in the severity of dysphagia, chest pain, regurgitation, or weight loss.^21^ Based on these findings, the relationship between IEM and symptoms remains unclear, and the effect of treatment on symptoms cannot be predicted.^21^^-^^23^

In the present study, most patients with IEM and pathological reflux had normal endoscopic findings. Grade B esophagitis was the second most common endoscopic finding in this group. In contrast, all patients with physiological reflux had normal endoscopic examinations findings. No significant differences in endoscopic findings were observed between the 2 groups. Additionally, hiatal hernia was present in approximately 30% of patients with IEM, with no significant difference between the groups. These findings suggest no association between endoscopic findings or hiatal hernia and acid exposure.

HRM analysis showed that type I was the most common EGJ morphology and that the mean LESP was 12.36 mm Hg in patients with IEM. Mean LESP and type III EGJ morphology differed significantly between the pathological and physiological reflux groups (*P* = .017 and *P* = .019, respectively). No significant differences were observed between the 2 groups in the median IRP, mean DCI, EGJ-CI, and peristalsis classification. Similarly, Jiang et al^24^ reported that type I (66.7%) was the most common EGJ morphology and that the mean LESP was 16 mm Hg in patients with IEM. Although the mean LESP was lower than that in patients with normal esophageal motility, the difference was not statistically different.

Acid exposure is influenced by transient lower esophageal sphincter relaxations (TLESRs) controlled by the efferent vagus nerve, esophageal dysmotility with impaired clearance, and structural abnormalities of the EGJ.^25^ The effectiveness of the EGJ is determined by LESP and CD contraction. EGJ contraction is regarded as a key defense against GERD. LESP, EGJ morphology, and EGJ-CI are important determinants of the EGJ barrier function. The differences between pathological and physiological reflux groups do not necessarily indicate a direct causal relationship with reflux but may reflect functional differences associated with reflux. LESP, EGJ morphology, and EGJ-CI are important determinants of EGJ barrier competence.

Pathological acid exposure is not determined solely by EGJ barrier function. Because EGJ-CI alone does not reliably predict pathological acid exposure, establishing cutoff values is unlikely to be clinically used as a standalone diagnostic tool. Moreover, no validated EGJ-CI cutoff value for predicting pathological acid exposure has been established. Some studies have suggested that no threshold value can be established with both high sensitivity and specificity.^26^ Conversely, Tolone et al proposed an EGI-CI cutoff range of 5-10, accepting lower specificity (54-63%) to achieve higher sensitivity (74-89%).^27^ Meanwhile, Ham et al^28^ reported an EGJ-CI cutoff of 30, with a sensitivity of 77.8% and a specificity of 81.7%; however, their findings were based on a sample of only 137 patients.

The Lyon Consensus v1.0 also identified methodological inconsistencies in EGJ-CI measurement and emphasized the need for further investigation. Subsequent studies have sought to address these issues^28^^-^^31^, and EGJ-CI was incorporated into the latest version of the CC v4.0. Nevertheless, the Lyon Consensus v2.0 has not yet provided updated recommendations or criteria for the use of EGJ-CI.

In this study, EGJ-CI did not differ significantly between the pathological and physiological reflux groups. In contrast, Reddy et al reported that EGJ-CI was significantly lower in patients with IEM than in those with normal esophageal motility (*P* < .01).^5^ Babbar et al reported that EGJ-CI was significantly lower in patients with pathological reflux than in those with nonpathological reflux.^32^ These findings support the importance of EGJ in barrier competence. A low EGJ-CI is an important predictor of acid exposure. In patients with IEM, reduced EGJ-CI may contribute to impaired EGJ barrier function and increased acid exposure. However, the absence of a significant difference in EGJ-CI between the pathological and physiological reflux groups may reflect the multifactorial nature of IEM acid exposure, the pathogenesis of pathological reflux in IEM, and EGJ barrier competence. EGJ contraction is considered a major defense against GERD. The competence of the EGJ is determined by LES pressure and CD contraction.^28^ EGJ-CI is considered an adjunctive or supportive metric for the assessment of GERD.

In the current study, although hiatal hernia was identified endoscopically in 30.4% of patients with IEM, EGJ-CI did not differ between pathological and physiological reflux groups. However, manometric EGJ morphology differed between the groups. Based on these findings, EGJ morphology may be better than endoscopy for detecting hiatal hernia associated with esophageal acid exposure. Li et al^33^also published a meta-analysis showing that HRM has better diagnostic performance than endoscopy and x-ray for detecting hiatal hernia.

According to the MII‑pH findings, the pathological reflux group had significant higher DeMeester score, AET (%), upright AET (%), supine AET (%), BET (%), upright BET (%), supine BET (%), total reflux number, long (>5 minutes) reflux episodes, longest reflux time, acid reflux episodes, total reflux episodes, and MNBI at 3 cm (Ω) than the physiological reflux group (supine BET (%) *P* = .014, total reflux episodes *P* = .004, and others *P* ≤ .001). Although acid reflux episodes and total reflux episodes were higher in the pathological reflux group, no significant differences were observed between the 2 groups in weakly acidic and nonacid reflux episodes. Chen et al^34^ reported that AET and total reflux episodes were significantly higher in patients with IEM group than in those with normal esophageal motility.

IEM is the most common motility disorder associated with GERD.^35^ A study examining the prevalence of GERD in patients with IEM and the influence of IEM on GERD development found that patients with IEM are more prone to acid reflux when not receiving PPIs and nonacid reflux while receiving PPIs. Reflux burden was higher in patients with IEM who had low lower LESP, type III EGJ, and a higher proportion of impaired swallowing.^5^ A notable difference in MNBI suggests that impaired esophageal clearance contributes to pathological reflux. Esophageal chemical clearance and mucosal integrity are mainly related to esophageal peristalsis. You HS et al^36^ found that MNBI values were higher in patients without IEM compared with those with IEM (*P* < .05).

Li Y et al^37^ evaluated the MNBI characteristics in patients with refractory GERD with different esophageal dynamics. The investigators compared 4 groups: patients with only an abnormal EGJ barrier, only esophageal body peristaltic abnormalities, combined EGJ barrier and esophageal peristaltic abnormalities, and normal EGJ barrier and esophageal peristalsis. They found that MNBI was lower in patients with both EGJ barrier and esophageal peristaltic abnormalities than in those with only abnormal EGJ barrier and those with normal EGJ barrier and esophageal peristalsis (*P* = .049 and *P* = .001, respectively). No significant differences were observed in other groups.

This study had several limitations. First, it was conducted at a single center with a limited sample size, which may have increased the risk of a type II error and limited the ability to draw conclusions regarding predictive performance. Second, symptom severity was not assessed. Symptom assessment was limited to the presence or absence of symptoms, and the lack of validated severity scores may have reduced sensitivity. Therefore, larger prospective studies with adequate numbers of participants in each subgroup are needed to confirm these findings. Future studies incorporating symptom severity scores should improve understanding of the clinical features of patients with IEM. Third, 24-hour MII-pH monitoring has limited sensitivity for detecting pathological acid reflux. Lastly, the authors did not analyze the manometric subtypes of IEM (nonsevere and severe) or contraction reserve. In patients with nonsevere IEM, upright and total AET were significantly higher in the absence of a contraction reserve. Conversely, no differences were observed between patients with and without contraction reserve in the severe IEM group.^38^Despite these limitations, the study demonstrated that pathological reflux symptoms are common in patients with IEM. Additionally, the authors found that mean LESP and EGJ morphology are predictive HRM metrics for pathological reflux in patients with IEM.

In conclusion, pathological reflux and other symptoms are common in patients with IEM. However, these symptoms are not solely attributable to pathological reflux. In addition to the weak and failed peristalsis characteristic of IEM, impaired esophageal clearance and EGJ barrier dysfunction contribute to acid exposure and symptoms. Nonetheless, HRM and MII-pH metrics should be regarded as markers associated with, rather than directly causes of, pathologic reflux. Future studies including a larger number of patients and comparisons of symptom severity are needed to further clarify the clinical implications of acid exposure in patients with IEM.

## Figures and Tables

**Table 1.** Demographic, Clinical, and Endoscopic Characteristics of IEM Patients

	All Patients (n = 69)	Pathological Reflux (n = 49)	Nonpathological Reflux (n = 20)	*P*
Age (years)	47.42 ± 16.14	48.83 ± 15.88	44.25 ± 17.08	.301
Sex				
Male, n (%)	29 (42)	23 (46.9)	6 (30)	.199
Female, n (%)	40 (58)	26 (53.1)	14 (70)	
BMI (kg/m^2^), mean ± SD	27.12 ± 5.28	27.89 ± 5.10	25.22 ± 5.36	.056
Smoking, n (%)	29 (42)	20 (40.8)	9 (45)	.751
Symptoms				
Regurgitation, n (%)	52 (75.4)	35 (71.4)	17 (85)	.239
Heartburn, n (%)	54 (78.3)	37 (75.5)	17 (85)	.389
Chest pain, n (%)	40 (58)	26 (53.1)	14 (70)	.199
Dysphagia, n (%)	41 (59.4)	29 (59.2)	12 (60)	.950
Cough, n (%)	38 (55.1)	29 (59.2)	9 (45)	.286
Belching, n (%)	37 (53.6)	25 (51.0)	(58.3)	.501
Nausea, n (%)	31 (44.9)	22 (44.9)	9 (45)	.994
Vomiting, n (%)	26 (37.7)	20 (40.8)	6 (30)	.404
Globus sensation, n (%)	28 (40.6)	20 (40.8)	8 (40)	.950
Hiccups, n (%)	26 (37.7)	19 (38.8)	7 (35)	.771
Dyspepsia, n (%)	51 (73.9)	36 (73.5)	15 (75)	.896
Epigastric pain, n (%)	40 (58)	30 (61.2)	10 (50)	.395
Endoscopy				.071
Normal, n (%)	58 (84.1)	38 (77.6)	20 (100)	
Grade A esophagitis, n (%)	2 (2.9)	2 (4.1)		
Grade B esophagitis, n (%)	6 (8.7)	6 (12.2)		
Grade C esophagitis, n (%)	2 (2.9)	2 (4.1)		
Grade D esophagitis, n (%)	1 (1.4)	1 (2.0)		
Hiatal hernia, n (%)	21 (30.4)	17 (34.7)	1 (5)	.232

BMI, body mass index; IEM, ineffective esophageal motility.

**Table 2.** HRM Metrics of IEM Patients

	All Patients (n = 69)	Pathological Reflux (n = 49)	Physiological Reflux (n = 20)	*P*
Mean LESP (mm Hg)	12.36 ± 5.88	11.14 ± 4.80	16.4 ± 8.3	**.017**
Median IRP (mm Hg)	3.60 ± 2.68	3.45 ± 2.46	3.5 ± 2.8	.677
Mean DCI (mm Hg.s.cm)	313.4 ± 112.3	303.75 ± 108.50	293.7 ± 100.1	.471
EGJ-CI (mm Hg.s.cm)	8.51 ± 16.63	9.51 ± 19.25	6.2 ± 6.4	.822
EGJ morphology (n [%])				**.046**
Type I	35 (50.7)	22 (44.9)	13 (65.0)	.133
Type II	13 (18.8)	8 (16.3)	5 (25.0)	.407
Type III	21 (30.4)	19 (38.8)	2 (10.0)	**.019**
Peristalsis classification				
% intact contractions	10.24 ± 14.25	10.34 ± 13.99	6.67 ± 10.73	.988
% weak contractions	44.86 ± 27.71	41.09 ± 28.10	54 ± 24.36	.080
% failed contractions	42.71 ± 34.68	47.00 ± 36.38	34.17 ± 27.78	.108
% fragmented contractions	1.15 ± 5.33	0.78 ± 4.49	0.83 ± 2.88	.371

Bold values indicate *P* < .05.

DCI, distal contractile integral; EGJ, esophagogastric junction; EGJ-CI, esophagogastric junction contractile integral; IEM, ineffective esophageal motility; IRP, integrated relaxation pressure; HRM, high-resolution manometry; LESP, lower esophageal sphincter pressure.

**Table 3.** MII-pH Metrics of IEM Patients

	All Patients (n = 69)	Pathological Reflux (n = 49)	Physiological Reflux (n = 20)	*P*
DeMeester score	32.96 ± 24.17	42.06 ± 21.18	6.45 ± 5.10	**<.001**
AET (%)	9.11 ± 7.21	11.79 ± 6.40	1.30 ± 1.20	**<.001**
Upright AET (%)	10.36 ± 8.85	13.38 ± 8.27	1.55 ± 1.58	**<.001**
Supine AET (%)	7.50 ± 8.52	9.76 ± 8.78	0.91 ± 1.27	**<.001**
BET (%)	4.11 ± 4.76	5.08 ± 5.14	1.28 ± 1.09	**<.001**
Upright BET (%)	6.30 ± 6.47	7.50 ± 6.63	2.80 ± 4.61	**<.001**
Supine BET (%)	1.66 ± 3.39	2.06 ± 3.85	0.50 ± 0.44	**.014**
Long (>5 minutes) reflux episodes	4.26 ± 4.23	5.46 ± 4.25	0.75 ± 0.96	**<.001**
Longest reflux time, minutes	18.57 ± 17.65	23.16 ± 18.08	5.21 ± 5.47	**<.001**
Acid reflux episodes, n	44.94 ± 31.02	55.69 ± 28.34	13.58 ± 10.04	**<.001**
Weak acid reflux episodes, n	43.70 ± 56.77	47.26 ± 63.87	33.33 ± 26.71	.591
Nonacid reflux episodes, n	0.81 ± 3.94	1.06 ± 4.55	0.08 ± 0.28	.796
Total reflux episodes, n	85.70 ± 68.87	98.40 ± 74.26	48.67 ± 27.93	**.003**
MNBI at 3 cm (Ω)	1.82 ± 0.94	1.44 ± 0.66	2.93 ± 0.74	**<.001**

Data presented as mean ± SD. Bold values indicate *P* < .05.

AET, acid exposure time; BET, bolus exposure time; IEM, ineffective esophageal motility; MNBI, mean nocturnal baseline impedance.

**Table 4.** Logistic Regression Analysis of Predictive Factors for Pathological Reflux

	OR	95% CI	*P*
Hiatal hernia	2.125	0.613-7.36	.235
Mean LESP	0.877	0.793-0.969	**.010**
Median IRP	0.931	0.770-1.127	.465
Mean DCI	0.998	0.993-1.003	.354
EGJ-CI	1.018	0.971-1.068	.456
EGJ morphology	2.026	1.026-4.004	**.042**
BET	1.347	1.601-9.237	**.003**
MNBI	0,099	0.028-0.349	**<.001**

Bold values indicate *P* < .05.

BET, bolus exposure time; CI, confidence interval; DCI, distal contractile integral; EGJ, esophagogastric junction; EGJ-CI, esophagogastric junction contractile integral; IRP, integrated relaxation pressure; LESP, lower esophageal sphincter pressure; MNBI, mean nocturnal baseline impedance; OR, odds ratio.

## Data Availability

The data that support the findings of this study are not available.
